# Adult attention-deficit/hyperactivity disorder traits in healthy adults associated with brain volumetric data identify precuneus involvement in traffic crashes

**DOI:** 10.1038/s41598-023-49907-3

**Published:** 2023-12-18

**Authors:** Handityo Aulia Putra, Kaechang Park, Hikaru Oba, Fumio Yamashita

**Affiliations:** 1 Research Organization for Regional Alliance, Kochi University of Technology, 185 Miyanokuchi Tosayamada‑cho, Kami, Kochi 782‑0003 Japan; 2 Graduate School of Health Sciences, Hirosaki University, 66‑1, Hon‑cho, Hirosaki, Aomori 036‑8564 Japan; 3 Division of Ultrahigh Field MRI, Institute for Biomedical Sciences, Iwate Medical University, 1‑1‑1 Idaidori, Yahaba‑cho, Shiwa‑gun, Iwate, 028‑3694 Japan

**Keywords:** Neuroscience, Psychology and behaviour

## Abstract

This large-scale study including 2548 healthy adults with no clinical attention-deficit/hyperactivity disorder (ADHD) diagnosis intended to clarify the complex relationships between cerebral grey matter volumes (GMVs), ADHD traits, and driving safety behaviours. Path analysis of magnetic resonance imaging (MRI) results and questionnaires about ADHD traits and traffic crashes over the past decade revealed significant correlations of ADHD traits with different brain regions relevant to different cognitive functions. The left precuneus responsible for visuospatial cognition was the sole region correlated with all ADHD trait categories, suggesting it plays an important role in understanding driving safety and traffic crashes. For the first time, a strong relationship was found among regional GMVs, ADHD traits, and real-life traffic crashes. These insights into the complex interplay may inform the development of an effective intervention with MRI examination to prevent traffic crashes. Large-scale brain volumetric data may further open social applications of behaviour science and neuroimaging.

## Introduction

Human behaviours are multifaceted and influenced by a multitude of external factors, such as human relationships^1^ and living environments^2^, as well as internal factors, including physical health^3^, mentality^4^, and personality traits^5^. Regardless of the factors at play, the assessment of human behaviours presents a challenge due to their high complexity and the low quantity of quantifiable data. In the specific domain of automobile operation, however, human behaviours may be evaluated with a higher degree of accuracy and reproducibility. This is due to the abundance of speed and location data available for analysis, as well as the clear and measurable consequences of behaviours in the form of traffic crashes. Physical disability^6^, mental health disorders^7^, and aggressive or violent character traits^8^ have all been linked to traffic incidents. In Japan, road rage and tailgating have become considerable social issues, leading to the implementation of legal regulations in 2022^9^. The driving environment may serve to amplify and highlight certain human behaviours, providing a unique opportunity for behavioural analysis and intervention.

Attention-deficit/hyperactivity disorder (ADHD) is a prevalent neurodevelopmental disorder affecting both children and adults. It is characterized by symptoms such as difficulty sustaining attention, hyperactivities including a sense of restlessness, and issues of self-control, which are often evident in behaviours of impatience or hasty decision-making^10,11^. These symptoms are closely reflected in the Adult ADHD Self-Report Scale (ASRS) by Kessler et al.^12^, a commonly used tool for identifying ADHD in adults. Three categories of ADHD symptoms, i.e., inattention, hyperactive motor, and hyperactive verbal, can be scored using the ASRS^13^. Many studies have demonstrated that individuals with ADHD are more likely to be involved in traffic crashes^14,15^. Another study reported that people with self-reported ADHD are prone to improper braking or stopping^16^, which may lead to traffic crashes. Emotion dysregulation, a recognized aspect of ADHD often reported by individuals completing the ASRS, can exacerbate the aforementioned ADHD symptoms. This heightened emotional state can potentially contribute to traffic crashes by impairing important driving safety behaviours (DSBs), such as adherence to speed limits, smooth and controlled steering, maintaining a safe following distance, and conducting regular safety checks^17^. It is estimated that < 2.5% of adults have ADHD^18,19^. However, many individuals who have not been diagnosed with ADHD display behaviours indicative of ADHD according to the ASRS, regardless of symptom severity. These individuals often drive, unaware of these behaviours. Investigating the factors contributing to problematic driving in this population can not only enhance the current understanding of the impact of ADHD-like behaviours on driving performances but also provide valuable insights for developing interventions aimed at preventing traffic crashes.

Several studies using magnetic resonance imaging (MRI) have shown that ADHD is associated with changes in grey matter volumes (GMVs) within the brain, particularly within the prefrontal cortex, corpus striatum, and cerebellum^20^. Adult patients with ADHD display altered GMVs in the frontal-striatal and frontal-parietal circuits^21^. Moreover, a consistent pattern of global reduction in GMVs has been observed in patients with ADHD, with distinct localization in the right lentiform nucleus extending to the caudate nucleus^22^. Despite these findings, the study of GMVs in healthy adults with ADHD tendencies is substantially under-researched^23^. Regarding the relationships between GMVs and driving performance, the existing research is rather limited. Previous studies typically used questionnaires to assess driving performance or conducted real-world driving experiments at crossroads. However, these studies were often limited by small sample sizes of < 50 participants, impeding comprehensive analyses of the complex nature of human behaviours^24^. Some studies have started to explore the relationship between regional GMV (rGMV) and driving safety. For instance, Yamamoto et al.^25^ discovered that rGMV was indicative of a high risk of unsafe driving in healthy older people. Additionally, Sakai et al.^26^ found that regional frontal GMV associated with executive function capacity may act as a risk factor for vehicle crashes in normal ageing adults. These findings suggest that further research is needed to fully understand the complex interactions between brain structure, ADHD symptoms, and driving behaviours.

In a more recent functional MRI (fMRI) study by a Honda research team, the connection between brain functional regions and eye-gaze movement was investigated using an fMRI-compatible driving simulator equipped with an eye-tracking device^27^. The study identified significant activity in the left precuneus and the right supplemental motor cortex (SMC) and lingual gyrus correlated with the gaze movement toward hazardous objects displayed on the driving simulator's monitor. The precuneus, which responded fastest in this experiment, is renowned for its engagement in various cognitive functions such as episodic memory^28^, visual imaging^29^, and spatial judgment^30^. Although this experiment was conducted in a virtual environment created by a driving simulator, these authors hypothesized that the left precuneus plays a pivotal role in driving safety performance under real-world conditions.

**Figure 1:**
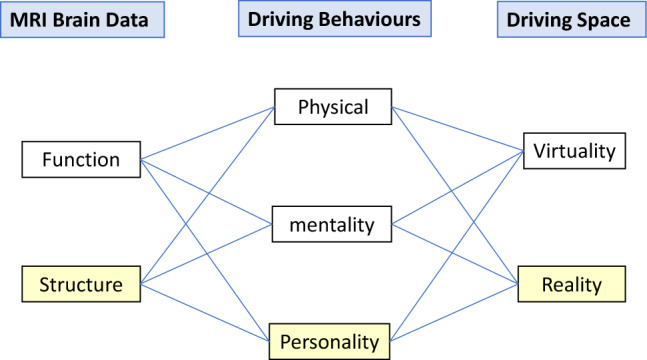
Components of brain magnetic resonance imaging data, driving behaviours, and driving space. The light-yellow squares indicate aspects investigated in the present study.

In a previous work, a significant correlation was found between the volume of the right SMC and the degrees of both physical and mental fatigue in healthy middle-aged adults^31^. This observation was based on a large-scale analysis of GMV data collected during Brain Dock, which has been developed and prevalent in Japan as MRI-based brain health check-ups^32^. This screening is uniquely implemented in Japan to facilitate the early detection of unruptured cerebral aneurysms^33^. Physical and mental fatigue are estimated through a questionnaire related to behaviours and psychological states indicative of tiredness or exhaustion^34^. We hypothesized that the relationship between ADHD symptoms and traffic crashes might also be illuminated by analysing GMV data from this extensive MRI dataset. In the current study, 2,548 healthy adults aged 23–76 years, who had no clinical diagnosis of ADHD, underwent MRI examinations and completed questionnaires about their ADHD symptoms and traffic crashes including near misses over the past 10 years. To the best of our knowledge, no other large-scale study has investigated the relationship between ADHD symptoms and actual traffic crashes using rGMV data, including those of the precuneus. The analysis used structural data obtained with a 1.5-T MRI, which is more common in medical practice than the 3-T MRI typically used for functional data analyses. The study design is depicted in Fig. 1.

## Results

**Figure 2:**
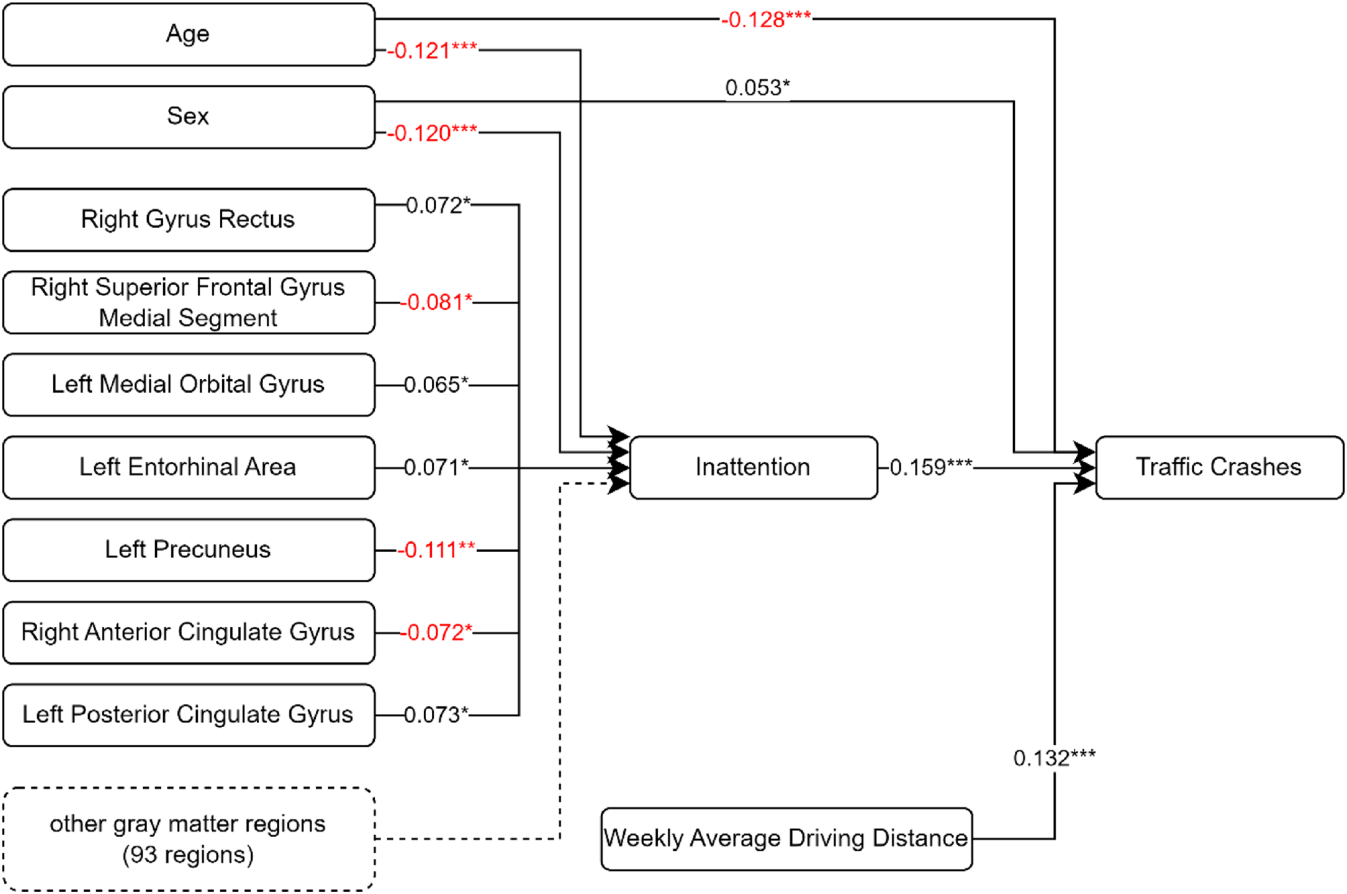
Path diagram of the path analysis with estimated effect values. The path considers the indirect effects of the 100 grey matter regions on traffic crashes through the attention-deficit/hyperactivity disorder category inattention. Straight lines: significant effects; dotted line: non-significant effect. *p < 0.05, **p < 0.01, ***p < 0.001.

In this study, the relationships between rGMV, inattention, hyperactive motor symptoms, hyperactive verbal symptoms, and traffic crashes were examined using path analysis. An adequate fit to the data was demonstrated using a model describing the relationships between rGMV, inattention, and vehicle crashes, with a chi-square value of 300.135 and 201 degrees-of-freedom (CMIN/DF = 1.493). The goodness-of-fit index (GFI = 0.998), comparative fit index (CFI = 0.999), and Tucker-Lewis index (TLI = 0.985) indicated an excellent fit of the model to the data. Evidence of a good model fit was also provided by the root mean square error of approximation (RMSEA = 0.014, 90% confidence interval [CI] [0.011, 0.017]). Seven grey matter regions including the right gyrus rectus (standardized path coefficient = 0.072, p < 0.05), right superior frontal gyrus medial segment (standardized path coefficient = −0.081, p < 0.05), left medial orbital gyrus (standardized path coefficient = 0.065, p < 0.05), left entorhinal area (standardized path coefficient = 0.071, p < 0.05), left precuneus (standardized path coefficient = −0.111, p < 0.01), right anterior cingulate gyrus (standardized path coefficient = −0.072, p < 0.05), and left posterior cingulate gyrus (standardized path coefficient = 0.073, p < 0.05) were associated with inattention tendency. Age (standardized path coefficient = −0.128, p < 0.001), sex (standardized path coefficient = 0.053, p < 0.05), and ADHD-characteristic inattention tendency (standardized path coefficient = 0.159, p < 0.001) were also associated with traffic crashes. The simplified path diagram for inattention is shown in Fig. 2.

**Figure 3:**
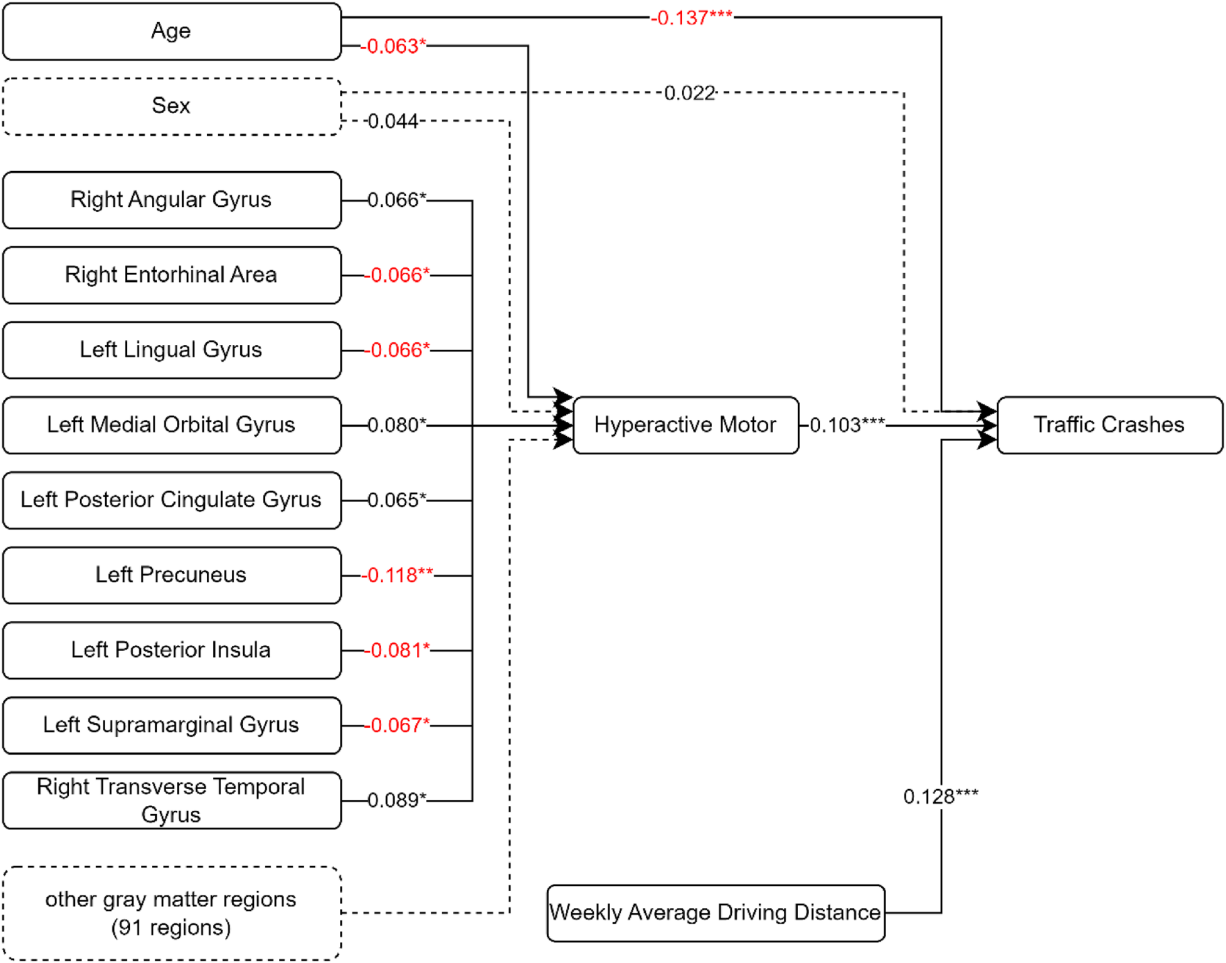
Path diagram of the path analysis with estimated effect values. The path considers the indirect effects of the 100 grey matter regions on traffic crashes through the attention-deficit/hyperactivity disorder category hyperactive motor. Straight lines: significant effects; dotted lines: non-significant effects. *p < 0.05, **p < 0.01, ***p < 0.001.

A similar pattern was observed for the model examining the relationship among rGMV, hyperactive motor symptoms, and traffic crashes, with an acceptable fit to the data demonstrated by a chi-square value of 303.666 and 201 degrees-of-freedom (CMIN/DF = 1.511). GFI (0.998), CFI (0.999), and TLI (0.984) suggested an excellent fit of the model to the data. RMSEA (0.014, 90% CI [0.011, 0.017]) also provided evidence of a good model fit. Nine GMV regions including the right angular gyrus (standardized path coefficient = 0.066, p < 0.05), right entorhinal area (standardized path coefficient = -0.066, p < 0.05), left lingual gyrus (standardized path coefficient = −0.066, p < 0.05), left medial orbital gyrus (standardized path coefficient = 0.080, p < 0.05), left posterior cingulate gyrus (standardized path coefficient = 0.065, p < 0.05), left precuneus (standardized path coefficient = -0.118, p < 0.01), left posterior insula (standardized path coefficient = −0.081, p < 0.05), left supramarginal gyrus (standardized path coefficient = -0.067, p < 0.05), and right transverse temporal gyrus (standardized path coefficient = 0.089, p < 0.05) were associated with hyperactive motor tendency. Age (standardized path coefficient = −0.137, p < 0.001) and ADHD-characteristic hyperactive motor tendency (standardized path coefficient = 0.103, p < 0.001) were associated with vehicle accidents. The simplified path diagram for hyperactive motor is shown in Fig. 3.

**Figure 4:**
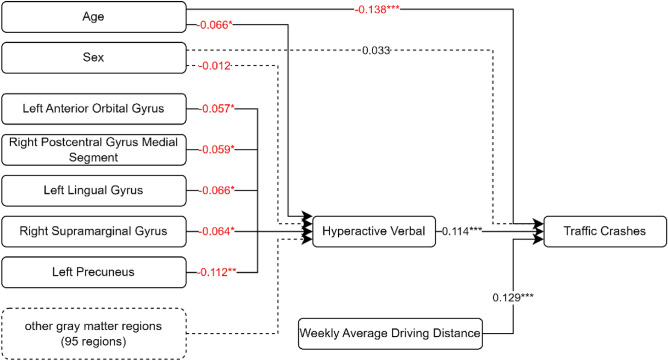
Path diagram of the path analysis with estimated effect values. The path considers the indirect effects of the 100 grey matter regions on traffic crashes through the attention-deficit/hyperactivity disorder category hyperactive verbal. Straight lines: significant effects; dotted lines: non-significant effects. *p < 0.05, **p < 0.01, ***p < 0.001.

Lastly, the model investigating the associations among rGMV, hyperactive verbal symptoms, and traffic crashes also demonstrated a good fit to the data, with a chi-square value of 305.082 and 201 degrees-of-freedom (CMIN/DF = 1.518). GFI (0.998), CFI (0.999), and TLI (0.984) indicated an excellent fit of the model to the data, with RMSEA (0.014, 90% CI [0.011, 0.017]) also providing evidence of a good model fit. Five GMV regions including the left anterior orbital gyrus (standardized path coefficient = −0.057, p < 0.05), right postcentral gyrus medial segment (standardized path coefficient = −0.059, p < 0.05), left lingual gyrus (standardized path coefficient = −0.066, p < 0.05), right supramarginal gyrus (standardized path coefficient = −0.064, p < 0.05), and left precuneus (standardized path coefficient = −0.112, p < 0.05) were associated with hyperactive verbal tendency. Age (standardized path coefficient = −0.138, p < 0.001) and ADHD-characteristic hyperactive verbal tendency (standardized path coefficient = 0.114, p < 0.001) were also associated with traffic crashes. The simplified path diagram for hyperactive verbal is shown in Fig. 4. Table 1 summarizes the estimated values of each rGMV in relation to ADHD symptoms. Additionally, the path estimates from each of the ADHD symptoms to traffic crashes are shown in Table 2.

**Table 1:** Path analysis estimates of the correlation between regional grey matter volumes and self-reported ADHD scores.

Grey matter regions	Inattention	H. Motor	H. Verbal
L. Basal forebrain	0.014	0.042	0.016
R. Basal forebrain	−0.044	0.014	−0.058
R. Anterior cingulate gyrus	−0.072*	−0.059	−0.054
L. Anterior cingulate gyrus	0.018	0.029	0.001
R. Anterior insula	0.041	−0.008	−0.016
L. Anterior insula	−0.061	−0.043	−0.029
R. Anterior orbital gyrus	−0.001	0.005	−0.018
L. Anterior orbital gyrus	0.02	−0.028	−0.057*
R. Angular gyrus	−0.008	0.066*	0.003
L. Angular gyrus	0.011	0.037	0.021
R. Calcarine cortex	−0.018	0.008	0.017
L. Calcarine cortex	0.005	0.007	0.026
R. Central operculum	−0.024	−0.027	−0.019
L. Central operculum	0.03	0.018	0.036
R. Cuneus	0.039	0.002	0.042
L. Cuneus	0.024	−0.001	−0.033
R. Entorhinal area	−0.046	−0.066*	−0.041
L. Entorhinal area	0.071*	0.06	0.056
R. Frontal operculum	0.022	0.05	0.032
L. Frontal operculum	0.006	0.018	0.016
R. Frontal pole	−0.033	0.059	0.027
L. Frontal pole	−0.009	0.008	0.01
R. Fusiform gyrus	−0.047	0.012	−0.04
L. Fusiform gyrus	−0.004	−0.032	−0.007
R. Gyrus rectus	0.072*	−0.014	−0.019
L. Gyrus rectus	−0.042	0.013	0.002
R. Inferior occipital gyrus	−0.014	0.015	−0.016
L. Inferior occipital gyrus	0.003	−0.005	−0.013
R. Inferior temporal gyrus	0.026	0.035	0.028
L. Inferior temporal gyrus	0.026	0.014	0.002
R. Lingual gyrus	0.027	0.028	0.036
L. Lingual gyrus	−0.035	−0.066*	−0.066*
R. Lateral orbital gyrus	−0.028	−0.028	−0.029
L. Lateral orbital gyrus	−0.021	−0.012	−0.019
R. Middle cingulate gyrus	0.021	0.034	0.009
L. Middle cingulate gyrus	−0.014	0.004	0.04
R. Medial frontal cortex	0.003	−0.04	−0.017
L. Medial frontal cortex	−0.017	−0.052	−0.039
R. Middle frontal gyrus	−0.067	−0.006	−0.01
L. Middle frontal gyrus	0.067	0.027	0.056
R. Middle occipital gyrus	0.005	−0.001	0.032
L. Middle occipital gyrus	−0.034	−0.039	−0.026
R. Medial orbital gyrus	−0.048	−0.043	0.043
L. Medial orbital gyrus	0.065*	0.08*	0.052
R. Postcentral gyrus medial segment	−0.035	−0.038	−0.059*
L. Postcentral gyrus medial segment	0.021	−0.01	0.05
R. Precentral gyrus medial segment	−0.003	−0.018	0.017
L. Precentral gyrus medial segment	−0.004	0.036	0.005
R. Superior frontal gyrus medial segment	−0.081*	−0.028	−0.028
L. Superior frontal gyrus medial segment	0.058	0.022	0.016
R. Middle temporal gyrus	0.06	0.004	0.022
L. Middle temporal gyrus	−0.03	−0.031	0.024
R. Occipital pole	0.041	0.019	−0.029
L. Occipital pole	−0.032	−0.028	−0.029
R. Occipital fusiform gyrus	−0.016	0.021	0.011
L. Occipital fusiform gyrus	0.01	−0.032	0.004
R. Opercular part of the inferior frontal gyrus	0.006	−0.025	−0.032
L. Opercular part of the inferior frontal gyrus	−0.02	0.029	−0.005
R. Orbital part of the inferior frontal gyrus	0.005	0.007	−0.051
L. Orbital part of the inferior frontal gyrus	0.035	−0.004	0.005
R. Posterior cingulate gyrus	−0.052	−0.026	−0.005
L. Posterior cingulate gyrus	0.073*	0.065*	0.018
R. Precuneus	0.051	0.046	0.048
L. Precuneus	−0.111**	−0.118**	−0.112**
R. Parahippocampal gyrus	0.006	0.023	0.023
L. Parahippocampal gyrus	−0.001	0.032	0.006
R. Posterior insula	−0.041	0.033	0.068
L. Posterior insula	−0.01	−0.081*	−0.062
R. Parietal operculum	−0.048	−0.049	−0.024
L. Parietal operculum	0.016	0.064	0.022
R. Postcentral gyrus	0.054	0.057	0.035
L. Postcentral gyrus	−0.026	−0.027	0.004
R. Posterior orbital gyrus	0.009	0.041	0.022
L. Posterior orbital gyrus	0.002	−0.003	0.001
R. Planum polare	0.038	−0.005	−0.009
L. Planum polare	0.003	0.016	0.008
R. Precentral gyrus	0.014	−0.013	−0.051
L. Precentral gyrus	−0.044	−0.021	−0.002
R. Planum temporale	−0.012	−0.038	−0.004
L. Planum temporale	−0.005	−0.016	−0.037
R. Subcallosal area	−0.007	−0.032	0.04
L. Subcallosal area	0.023	−0.017	0.028
R. Superior frontal gyrus	0.042	−0.036	−0.019
L. Superior frontal gyrus	0.003	−0.037	0.005
R. Supplementary motor cortex	0.04	0.02	−0.02
L. Supplementary motor cortex	−0.015	0.016	0.054
R. Supramarginal gyrus	0.027	0.014	−0.064*
L. Supramarginal gyrus	−0.033	−0.067*	−0.049
R. Superior occipital gyrus	0.027	0.024	−0.009
L. Superior occipital gyrus	0.002	0.036	0.012
R. Superior parietal lobule	0.04	0.002	0.037
L. Superior parietal lobule	−0.017	−0.008	0.002
R. Superior temporal gyrus	−0.011	0.001	−0.012
L. Superior temporal gyrus	0.022	0.013	0.041
R. Temporal pole	0.014	0.004	0.002
L. Temporal pole	−0.01	−0.035	−0.035
R. Triangular part of the inferior frontal gyrus	−0.005	0.003	0.019
L. Triangular part of the inferior frontal gyrus	−0.024	−0.047	−0.049
R. Transverse temporal gyrus	0.017	0.089*	0.018
L. Transverse temporal gyrus	−0.017	−0.037	−0.001
Age	−0.121***	−0.063*	−0.066*
Sex	−0.120***	0.044	−0.012

*L.* left, *R.* right, *H.* hyperactive.

*p < 0.05, **p < 0.01, ***p < 0.001.

**Table 2:** Path analysis estimates of the correlation between self-reported traffic crash records and ADHD symptoms, age, sex, and driving distance.

			Inattention	Hyperactive motor	Hyperactive verbal
Traffic crash	←	ADHD	0.159***	0.103***	0.114***
Traffic crash	←	Average weekly driving distance	0.132***	0.128***	0.129***
Traffic crash	←	Age	−0.128***	−0.137***	−0.138***
Traffic crash	←	Sex	0.053*	0.022	0.033

*ADHD* attention-deficit/hyperactivity disorder.

*p < 0.05, ***p < 0.001.

## Discussion

Our investigation shows that the left precuneus, a shared region among the three ADHD categories, may have a critical influence on how ADHD symptoms correlate with real-life traffic crashes (Fig. 2). This finding suggests that there is an association between reduced precuneus volume and impaired visual imagery and spatial judgment capabilities, which may be related to an increased likelihood of traffic crashes. Intriguingly, these results are in line with the findings of a previous fMRI study that highlighted the left precuneus as an essential element for DSBs through the monitoring of eye-gaze movements in virtual driving simulations^27^. This agreement between structural and functional data could mean that the observations derived from experimental virtual environments may apply to real-world traffic conditions. Over time, real-time functional brain responses may translate into significant structural alterations of the brain.

The lingual gyrus, a component of the visual cortex essential for word identification and recognition^35^, has been implicated in hyperactive motor and verbal traits in ADHD (Figs. 3 and 4). A previous fMRI study also reported the involvement of the right SMC and lingual gyrus, albeit with a lateral difference^27^. Although the SMC was not identified as a structure of interest in our current investigation, there appears to be a relationship between a reduced lingual gyrus volume and cognitive challenges, such as difficulties recognizing road signs.

A correlation between different regions of the brain and specific ADHD symptoms was also notable. For instance, in the context of inattention traits, we found significant associations with areas including the cingulate gyrus, entorhinal area, orbital gyrus, superior frontal gyrus, gyrus rectus, and precuneus (Fig. 3). The numbers presented in the graph represent the association direction. A positive number indicates a positive association, meaning that as regional brain volume increases, inattention also increases. Conversely, a negative number indicates a negative association, implying that as regional brain volume decreases, inattention increases. In the context of DSBs, the cingulate gyrus is involved in decision-making processes^36^, the entorhinal area serves as a widespread network hub for memory, navigation, and perception of time^37^, the orbital gyrus plays a role in the integration of emotions and memories linked with sensory experiences^38^, and the superior frontal gyrus is related to higher cognitive functions, particularly working memory^39^.

For the hyperactive motor category of ADHD symptoms, associations were newly found with the angular gyrus, posterior insula, supramarginal gyrus, and transverse temporal gyrus (Fig. 4). Regarding DSBs, the angular gyrus contributes to attention and spatial cognition^40^, the posterior insula aids in processing interoceptive information about the body's physiological status^41^, the supramarginal gyrus is involved in the perception of space and limb location^42^, and the transverse temporal gyrus is vital for processing incoming auditory information^43^.

**Figure 5:**
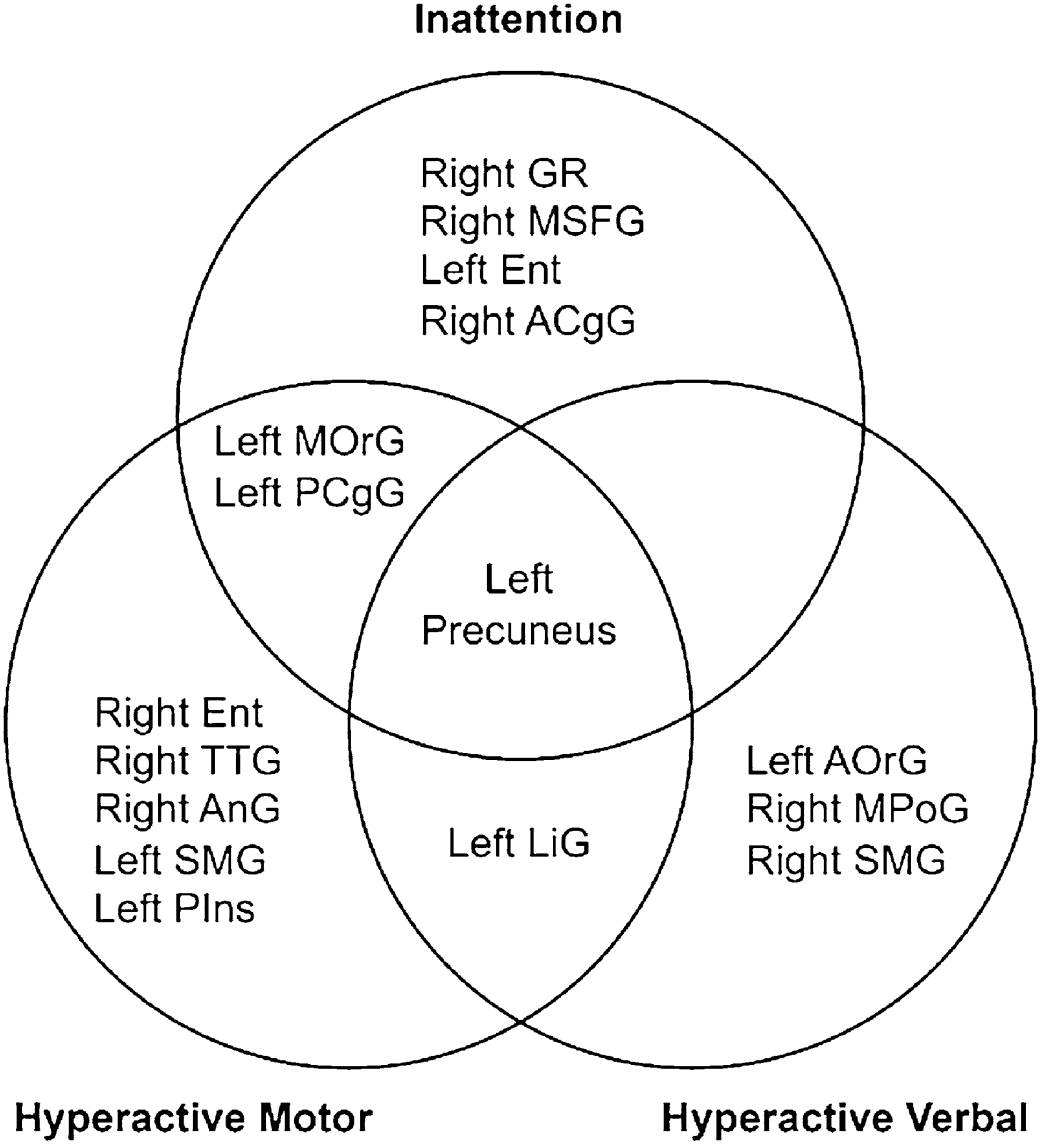
Venn diagram of grey matter regions that correlate with the attention-deficit/hyperactivity disorder categories inattention, hyperactive motor, and hyperactive verbal. *GR* gyrus rectus, *MSFG* superior frontal gyrus medial segment, *Ent* entorhinal area, *ACgG* anterior cingulate gyrus, *MOrG* medial orbital gyrus, *PCgG* posterior cingulate gyrus, *TTG* transverse temporal gyrus, *AnG* anterior gyrus, *SMG* supramarginal gyrus, *Pins* posterior insula, *LiG* lingual gyrus, *AOrG* anterior orbital gyrus, *MPoG* postcentral gyrus medial segment.

Regarding hyperactive verbal traits associated with ADHD, the postcentral gyrus has emerged as a linked area of interest (Fig. 5). According to recent research, this region of the brain is involved in sensorimotor integration, which is vital for coordinating and perceiving movement^44^. This function is critical, for instance, in scenarios requiring the judgement of the speed and direction of an approaching vehicle. Therefore, it is plausible that alterations in the postcentral gyrus may impact such judgements, potentially leading to heightened verbal hyperactivity symptoms seen in patients with ADHD.

**Figure 6:**
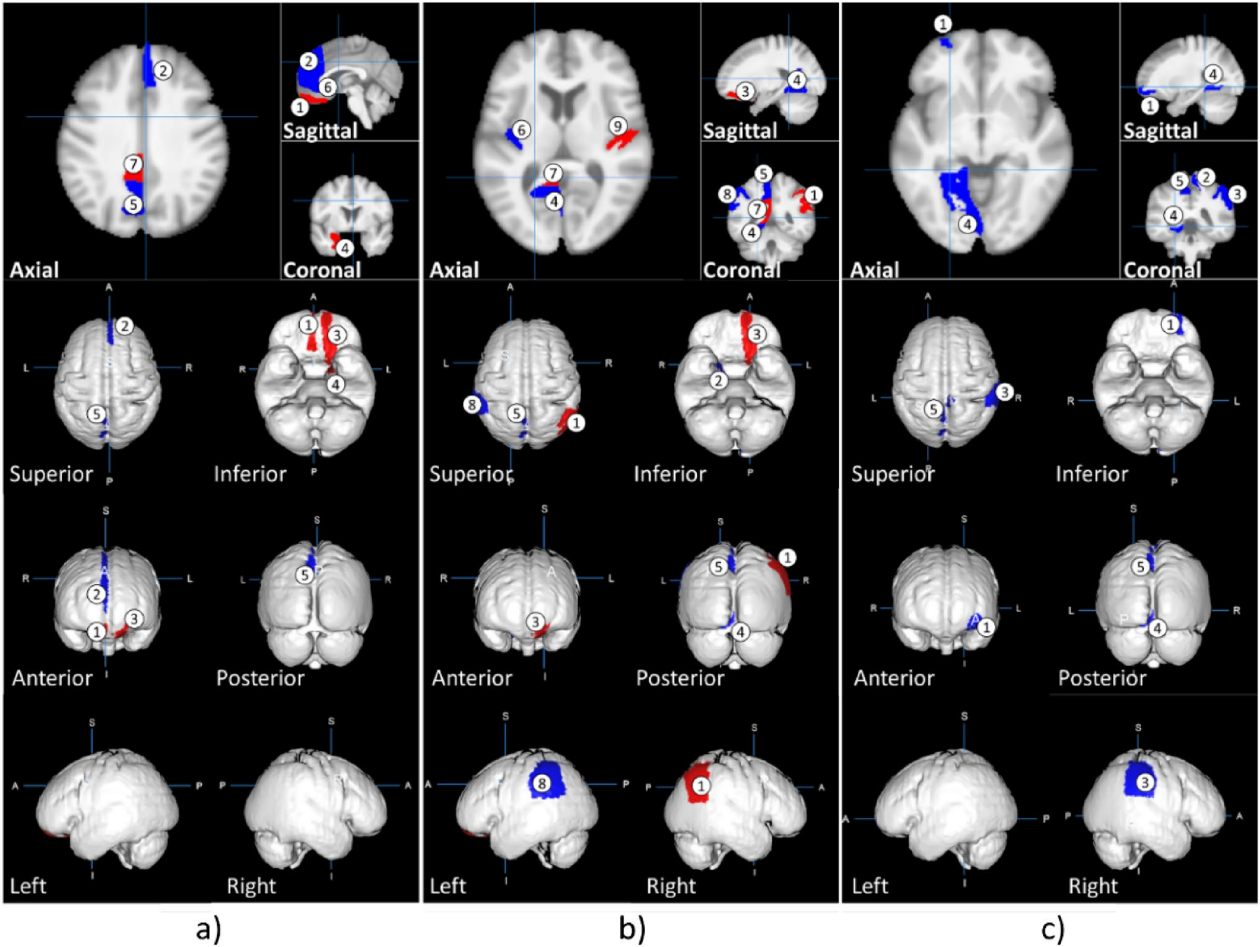
Illustration of significantly associated brain regions (see Table 1). Regions with estimated positive values are coloured in red and those with negative values in blue. The images illustrate the brain regions associated with inattention (**a**), hyperactive motor (**b**), and hyperactive verbal (**c**). The indicated brain regions for inattention are as follows: (a1) right gyrus rectus, (a2) right superior frontal gyrus medial segment, (a3) left medial orbital gyrus, (a4) left entorhinal area, (a5) left precuneus, (a6) right anterior cingulate gyrus, and (a7) left posterior cingulate gyrus. For hyperactive motor symptoms, the indicated brain regions are as follows: (b1) right angular gyrus, (b2) right entorhinal area, (b3) left medial orbital gyrus, (b4) left lingual gyrus, (b5) left precuneus, (b6) left posterior insula, (b7) left posterior cingulate gyrus, (b8) left supramarginal gyrus, and (b9) right transverse temporal gyrus. For hyperactive verbal behaviour, the corresponding brain regions include the following: (c1) left anterior orbital gyrus, (c2) right postcentral gyrus medial segment, (c3) right supramarginal gyrus, (c4) left lingual gyrus, and (c5) left precuneus.

DSBs necessitate the ability to focus and distribute attention among multiple sensory events via visual, auditory, spatial cognition, and others^45^. Subsequently, the interpretation of potentially hazardous situations, swift cognitive decision-making in complex and rapidly changing environments, and execution of appropriate driving manoeuvres are also required. Given these demands, it is unsurprising that numerous and diverse regions of grey matter are implicated (Fig. 6). However, the importance of the precuneus among grey matter regions cannot be overemphasized because it was the only region common to all examined ADHD categories and responded fastest to hazardous traffic situations that a Honda research team produced with fMRI-compatible driving simulators. The identification of the relationship between rGMVs including the precuneus and traffic crashes via ADHD symptoms might offer the identification of dangerous drivers with MRI volumetric data. In Japan, brain healthcare check-ups utilizing conventional 1.5-T MRI have been popular, and we already reported the significant associations of leukoaraiosis due to cerebral ischaemic lesions with traffic crashes^46,47^ and wrong-way entries on highways based on large-scale data of MRI healthcare check-ups^48^. Thus, large-scale brain structural data obtained by 1.5-T MRI may contribute to the reduction and preclusion of traffic crashes.

Our results showed both positive and negative GMV responses to ADHD inattention and hyperactive motor traits, with all regions associated with hyperactive verbal traits responding negatively. If these volumetric alterations represent hyperactivity or hypoactivity of neuronal functions, the mixed responses to inattention and hyperactive motor traits might indicate more intricate interactions between these grey matter regions. This aligns with previous research on fatigue, which demonstrated mixed positive and negative correlations between fatigue levels and rGMVs in healthy middle-aged adults^31^. In cases of chronic or pathological fatigue, rGMVs decrease^49^. Watanabe^50^ suggested that the initial stages of fatigue progression may initiate biological compensation to overcome fatigue and upregulate metabolism, resulting in increased volumes on positron emission tomography (PET) images. Generally, patients with ADHD exhibit thinner cortical thickness than neurotypical individuals^51,52^. However, some research findings indicate that the frontal cortices and caudate nucleus volumes of patients with ADHD can be larger^53,54^. This suggests that some of the study participants were in the early or mild stage of ADHD progression, where biological compensation is possible. To validate this hypothesis, metabolic analyses such as PET may be necessary.

The robust path analysis in our study demonstrating significant associations in our models offers valuable insights into the relationships between rGMV, ADHD symptoms, and traffic crashes. All examined models exhibited excellent fits, with the model examining the interplay among rGMV, inattention, and traffic crashes reporting an exceptional fit^54,55^. These values collectively indicate an excellent fit of the model to the data, with CFI and TLI values above 0.95 and RMSEA values below 0.06 generally being indicative of a well-fitting model^55^. Further models exploring the relationships between rGMV, hyperactive motor symptoms, hyperactive verbal symptoms, and traffic crashes also yielded acceptable model fit indices, which is a key requirement for valid inferences in path analysis^56^. The current model via ADHD tendencies may be statistically optimal, although not compared with a model analysing a direct relationship between rGMV and traffic crashes.

Our findings emphasize the importance of considering ADHD characteristics and regional volumetric data, especially of the precuneus, in understanding and potentially mitigating traffic crash risks. This study also accentuates the critical need for larger-scale brain structure data with 1.5-T MRI, which, although challenging to obtain but still easier than functional data with 3-T MRI, could shed further light on the complex interplay among brain structure, ADHD symptoms, and driving behaviour. Future research will need to overcome the limitations of our data collection method, which relied on self-reported data obtained by medical interviews during brain healthcare check-ups. Unfortunately, the Japanese National Police Agency does not open accident databases for academic research. A direct MRI investigation of drivers involved in traffic crashes worldwide is eagerly anticipated for data confirmation. Additionally, this study was conducted at a single centre in Kochi Prefecture in Japan, which represents a selection bias.

Finally, these findings suggest promising real-world applications. For instance, by incorporating a driving questionnaire that assesses ADHD symptoms based on MRI volumetric data during the driver's license renewal process, we can proactively identify individuals at a higher risk of causing traffic crashes. This approach would enable us to provide targeted safety guidance and interventions to these drivers, helping to prevent traffic accidents and enhance road safety. This is just one example of how our research can be harnessed for practical purposes, demonstrating its potential to make a meaningful impact in improving driver safety and reducing traffic-related incidents.

## Methods

### Participants

The study population consisted of 2,548 healthy participants (1,488 men and 1,060 women) with a mean age of 52.76 ± 8.632 years who lived in Kochi Prefecture, Japan. Participants undergoing brain healthcare check-ups in Kochi Kenshin Clinic affiliated with Kochi University of Technology were recruited. All participants had a Mini-Mental State Examination score of ≥ 28 and were right-handed. They answered a questionnaire regarding their ADHD symptoms and traffic crash history. A medical specialist (K.P.) conducted MRI. Additionally, participants with artifacts caused by dental treatment or cerebrovascular/neurological diseases such as cerebral infarction, high-graded leukoaraiosis, brain tumours, arachnoid cysts, hydrocephalus, and psychotic disorders such as schizophrenia were excluded because of their influence on rGMV measurements.

### Ethics statement

This study adhered to the Ethics Guidelines for Medical and Health Research Involving Human Subjects, based on the Declaration of Helsinki. All participants provided written informed consent before inclusion in the study, any questions regarding the study were addressed by the research team, and the study participants were informed that their personal information will be kept confidential. Additionally, participants were informed that their data will only be used for scientific purposes. All measurements were performed to ensure that the study complied with ethical standards, including respect for individuals’ autonomy, avoiding potential harm or discomfort, and preserving the confidentiality of all personal information. The data obtained from the study were kept confidential and used solely for research purposes. This study was approved by the institutional review board at Kochi University of Technology (application no. 122-C1).

### MRI image acquisition and measurement

In the present study, T1-weighted MRI was performed using the 1.5-T ECHELON Vega system (Hitachi, Tokyo, Japan) with a three-dimensional gradient echo with an inversion recovery sequence. The imaging protocol incorporated a repetition time of 9.2 ms, an echo time of 4.0 ms, an inversion time of 1,000 ms, a flip angle of 8°, a field of view of 240 mm, a matrix size of 0.9375 × 0.9375 mm, a slice thickness of 1.2 mm, and a single excitation. All images were thoroughly inspected for brain pathologies or anomalies, head motion, and artefacts that could influence volumetric measurements. The VBM8 toolbox (http://dbm.neuro.uni-jena.de/vbm/) and other modules in Statistical Parametric Mapping (SPM) 12 (https://www.fil.ion.ucl.ac.uk/spm/) were employed to calculate regional brain volumes. The acquired images were segmented into grey matter, white matter, and cerebrospinal fluid space using the maximum a posteriori approach^57^. The segmented grey and white matter images were further processed to establish morphological correspondence between the template image and the participant's brain using a high-dimensional non-linear warping algorithm^58^. The estimated non-linear warp was subsequently applied inversely to an atlas defined in the template space, enabling anatomical parcellation of the target brain. The neuromorphometric atlas included in SPM12 was utilized for parcellation, with adjustments for white matter lesions that appeared as incorrect grey matter segments around the lateral ventricles. Volumes for each anatomical region were estimated by summing the corresponding tissue densities in voxels belonging to the respective region. All volumes were normalized by the intracranial volume for each participant before statistical analysis.

### Questionnaire regarding traffic crashes

All participants were interviewed by a medical doctor (K.P.) during brain healthcare checkups and asked to complete a questionnaire regarding their driving habits, including their average weekly driving frequency, distance, and hours spent driving. Moreover, they were asked to recall their experiences with traffic crashes, including near crashes, at intersections in the past 10 years. The questionnaire is asked voluntarily and described as follows: Have you ever had head-on and/or rear-end collisions at intersections? Have you ever almost caused crashes at intersections? Each question required participants to indicate the number of traffic crashes or near-crashes they experienced. Intersection crashes are regarded as one of the most frequent accidents, involving inattentive or uncareful driving, although traffic crashes occur at various places or sites of the road networks.

### Questionnaire regarding ADHD symptoms

Participants in the study completed a questionnaire assessing ADHD symptoms using Part B of the ASRS^13^ during brain healthcare checkups. The questionnaire is asked voluntarily and the ASRS comprises two distinct parts: Part A, consisting of six questions aimed at diagnosing ADHD, and Part B, encompassing 12 questions specifically designed to measure ADHD symptoms.

In alignment with our research objectives, our study focused exclusively on Part B of the ASRS questionnaire. This section evaluates ADHD symptoms pertaining to inattention, hyperactive motor behaviour, and hyperactive verbal behaviour. Participants were instructed to rate the frequency of their experiences over the past 6 months using a five-point Likert scale, ranging from 0 ("never") to 4 ("very often"). The ASRS questionnaire has demonstrated robust reliability and validity in detecting ADHD symptoms among adults^12^. By emphasizing Part B, our aim was to examine ADHD symptomatology in a sample of healthy adult participants without a reported history of ADHD, enabling a focused investigation aligned with our research objectives.

### Statistical analysis

Separate path analyses were conducted for each ADHD category (inattention, hyperactive motor, and hyperactive verbal). The relationships between rGMV, ADHD category, and traffic crashes were assessed while controlling for age, sex, and average weekly driving distance as covariates. Analyses were conducted using IBM SPSS 29 and IBM AMOS 29 (IBM Corp., Armonk, NY, USA).

To better understand the complex relationships based on multiple variables, the present study employs path analysis, which is a statistical method to examine the direct and indirect effects of multiple variables and provide a more comprehensive understanding of the complex interplay between variables^59^. This approach enables the investigation of the multifaceted effects of age, sex, and rGMVs on traffic crashes (or near-crashes) according to the three examined categories of ADHD symptoms.

In our study, we initially conducted a path analysis involving 100 regional gray matter volume (rGMV) parameters. To navigate the complexity of our model and reduce the risk of Type I errors, we subsequently performed additional path analyses focused on the rGMV parameters that displayed significant associations. These significant rGMVs are illustrated in Fig. 2 for inattention (comprising 7 rGMVs), Fig. 3 for hyperactive motor symptoms (involving 9 rGMVs), and Fig. 4 for hyperactive verbal symptoms (encompassing 5 rGMVs).

To effectively address the increased number of comparisons and control the familywise error rate, we employed the Bonferroni correction. The Bonferroni correction is a conservative method that reduces the risk of Type I errors when conducting multiple comparisons. It involves dividing the original alpha level (α = 0.05) by the total number of comparisons conducted for each set. The corrected significance levels were set at α = 0.005 for inattention (encompassing 10 comparisons, including rGMVs, age, sex, and driving distance), α = 0.0042 for hyperactive motor symptoms (comprising 12 comparisons), and α = 0.0063 for hyperactive verbal symptoms (comprising 8 comparisons). We utilized these corrected alpha levels as thresholds to confirm the statistical significance of the presented rGMVs.

The model fit was confirmed using the following indicators: chi-square to degrees-of-freedom ratio (CMIN/DF), GFI, CFI, and TLI. A CMIN/DF of 1–3, a lower RMSEA (< 0.05), and higher GFI, CFI, and TLI values (> 0.90) indicate a better model fit.

Furthermore, it's essential to recognize that our chosen statistical technique, Path Analysis, comes with certain assumptions. These include the presumption of linear relationships between variables (rGMV, ADHD symptoms, and traffic crashes), the necessity for uncorrelated errors, the exogeneity of independent variables, and the avoidance of endogeneity within the model. These assumptions have been thoughtfully considered and incorporated into our analysis. However, it's important to acknowledge that the real relationships in the brain and behavior might be more intricate and nonlinear. Future research might explore models with additional variables and more relaxed assumptions to deepen our understanding of these complex relationships.

## Data Availability

The data supporting the findings of this study are available upon request from the corresponding author.
